# Physiological predictors of respiratory and cough assistance needs after extubation

**DOI:** 10.1186/s13613-018-0360-3

**Published:** 2018-02-05

**Authors:** Nicolas Terzi, Frédéric Lofaso, Romain Masson, Pascal Beuret, Hervé Normand, Edith Dumanowski, Line Falaize, Bertrand Sauneuf, Cédric Daubin, Jennifer Brunet, Djillali Annane, Jean-Jacques Parienti, David Orlikowski

**Affiliations:** 1 INSERM, Université Grenoble-Alpes, U1042, HP2, 38000 Grenoble, France; 2 CHU Grenoble Alpes, Service de réanimation médicale, 38000 Grenoble, France; 3 Service de réanimation médicale, Centre Hospitalier Universitaire Grenoble - Alpes, CS10217, Grenoble Cedex 09, France; 4 Université de Versailles Saint Quentin en Yvelines, INSERM U1179, Garches, France; 5 CIC 1429, INSERM, AP-HP, Hôpital Raymond Poincaré, 92380 Garches, France; 6 Service d’Explorations Fonctionnelles Respiratoires, AP-HP, Hôpital Raymond Poincaré, 92380 Garches, France; 7 Service de Réanimation, Centre Hospitalier de Roanne, 42300 Roanne, France; 8 INSERM, U1075, 14000 Caen, France; 9 Université de Caen, 14000 Caen, France; 10 CHRU Caen, Service d’Explorations Fonctionnelles Respiratoire, 14000 Caen, France; 11 INSERM U 1179, Université de Versailles-Saint Quentin en Yvelines, 104 Bd Raymond Poincaré, 92380 Garches, France; 12 CIC 1429, Inserm-APHP, Hôpital Raymond Poincaré, 104 Bd Raymond Poincaré, 92380 Garches, France; 13 Service de Réanimation Médicale Polyvalente, Centre Hospitalier Public du Cotentin, BP 208, 50102 Cherbourg-en-Cotentin, France; 14 General Intensive Care Unit, Raymond Poincaré Hospital (AP-HP), Laboratory of Inflammation and Infection, U1173, INSERM and University of Versailles SQY, 92380 Garches, France; 15 Unité de Biostatistique et de Recherche Clinique, Centre Hospitalier Universitaire de Caen, Avenue de la Côte de Nacre, 14033 Caen, France; 16 Pôle de ventilation à domicile, AP-HP, Hôpital Raymond Poincaré, 92380 Garches, France; 17 Service de Santé Publique, AP-HP, Hôpital Raymond Poincaré, 92380 Garches, France

## Abstract

**Background:**

Identifying patients at high risk of post-extubation acute respiratory failure requiring respiratory or mechanical cough assistance remains challenging. Here, our primary aim was to evaluate the accuracy of easily collected parameters obtained before or just after extubation in predicting the risk of post-extubation acute respiratory failure requiring, at best, noninvasive mechanical ventilation (NIV) and/or mechanical cough assistance and, at worst, reintubation after extubation.

**Methods:**

We conducted a multicenter prospective, open-label, observational study from April 2012 through April 2015. Patients who passed a weaning test after at least 72 h of endotracheal mechanical ventilation (MV) were included. Just before extubation, spirometry and maximal pressures were measured by a technician. The results were not disclosed to the bedside physicians. Patients were followed until discharge or death.

**Results:**

Among 3458 patients admitted to the ICU, 730 received endotracheal MV for longer than 72 h and were then extubated; among these, 130 were included. At inclusion, the 130 patients had mean ICU stay and endotracheal MV durations both equal to 11 ± 4.2 days. After extubation, 36 patients required curative NIV, 7 both curative NIV and mechanical cough assistance, and 8 only mechanical cough assistance; 6 patients, all of whom first received NIV, required reintubation within 48 h. The group that required NIV after extubation had a significantly higher proportion of patients with chronic respiratory disease (*P* = 0.015), longer endotracheal MV duration at inclusion, and lower Medical Research Council (MRC) score (*P* = 0.02, *P* = 0.01, and *P* = 0.004, respectively). By multivariate analysis, forced vital capacity (FVC) and peak cough expiratory flow (PCEF) were independently associated with (NIV) and/or mechanical cough assistance and/or reintubation after extubation. Areas under the ROC curves for pre-extubation PCEF and FVC were 0.71 and 0.76, respectively.

**Conclusion:**

In conclusion, FVC measured before extubation correlates closely with FVC after extubation and may serve as an objective predictor of post-extubation respiratory failure requiring NIV and/or mechanical cough assistance and/or reintubation in heterogeneous populations of medical ICU patients.

ClinicalTrials.gov as #NCT01564745

**Electronic supplementary material:**

The online version of this article (10.1186/s13613-018-0360-3) contains supplementary material, which is available to authorized users.

## Background

Weaning patients off endotracheal positive-pressure ventilation involves two steps: separation of the patient from the ventilator and extubation. The day of extubation is a critical time during an intensive care unit (ICU) stay, as extubation failure occurs in 10–20% of patients and is associated with up to 50% hospital mortality [1–6]. There is some evidence that extubation failure can directly worsen patient outcomes independently of underlying illness severity [5]. Several factors may contribute to extubation failure, including cough impairment and presence of thick and/or excessive mucus, in addition to hypoventilation [4]. Cough assistance and noninvasive mechanical ventilation (NIV) can help to prevent post-extubation respiratory failure. However, as these techniques are time-consuming, criteria for selecting those patients most likely to benefit would be useful. Ideally, these criteria would be objective, easily measured parameters obtained immediately before and/or after extubation. Adequate respiratory muscle strength is essential to generate the pressures and flows needed to clear airway secretions during coughing. Accordingly, peak cough expiratory flow (PCEF) was found in many studies to predict successful decannulation and extubation [7–12]. However, the tracheal tube can alter PCEF values via two mechanisms: it elevates airway resistance [13]; and it eliminates the role of the glottis in coughing [14].

Here, our objective was to evaluate the accuracy of parameters easily collected before versus after extubation in predicting the risk of post-extubation respiratory failure requiring, at best, NIV and/or mechanical cough assistance and, at worst, reintubation. We assessed cough performance and other easily collected respiratory parameters obtained before and after extubation, with the goal of determining which parameters and measurement conditions best identified patients who would require NIV and/or mechanical cough assistance after extubation.

## Methods

### Study population

We conducted a multicenter, prospective, observational study in two university-affiliated hospitals (Caen and Garches) and one general hospital (Roanne) in France from April 2012 through April 2015. The appropriate ethics committee (CPP Nord-Ouest III) approved the study (#2011-A00849-32), which was registered on ClinicalTrials.gov (#NCT01564745). All patients provided written informed consent.

Patients 18 years of age or older and sufficiently cooperative without sedation were eligible if they were admitted to the ICU and received invasive mechanical ventilation (MV) for at least 72 h then passed a weaning test performed according to recommendations [4, 15, 16]. Exclusion criteria were previous long-term NIV at home and unavailability of an lung function test (LFT) technician.

### Study procedures

Weaning from the ventilator was performed following a standardized protocol. Patients were screened daily for predefined weaning-readiness criteria, i.e., improvement in clinical signs, peripheral capillary oxygen saturation (SpO_2_) > 92% with fraction of inspired oxygen < 50% and positive end-expiratory pressure < 5 cm H_2_O, no infusion of vasopressor agents or sedatives, and adequate responses to simple commands. When these criteria were met, a spontaneous breathing test (SBT) was performed, by having the patient either breathe spontaneously from the ventilator on a T piece or receive pressure-support ventilation with an inspiratory pressure of 7 cmH_2_O and zero end-expiratory pressure. The test was interrupted if any of the following signs of poor tolerance was observed: respiratory rate > 35/min, SpO_2_ < 90%, heart rate > 140/min, and arterial systolic blood pressure > 180 mmHg or < 90 mmHg. Patients who successfully completed the test were considered for a trial of extubation. Decisions to perform a cuff-leak test and/or give corticosteroid therapy were based on standard practice at each study center.

Patients who passed an SBT and were considered for extubation underwent lung function testing (LFT) (see Additional file 1). After extubation, the patients breathed spontaneously with an oxygen flow titrated to maintain SpO_2_ > 90%.

Physicians were blinded to LFT results. Patients were followed until ICU discharge or death.

### Lung function testing (LFT)

LFT was repeated after extubation provided and there was no laryngeal edema (see Additional file 1).

### Clinical data

At ICU admission, we recorded the following: comorbidities, MV duration at inclusion, number of tracheal aspirates within 24 h before extubation, Glasgow Coma Scale score, Medical Research Council (MRC) scale combined score for muscle strength [17], and Borg Scale [18] score for subjective dyspnea.

### Extubation care and definitions

According to guidelines, patients were extubated by the physician if they passed an SBT [4, 15, 16]. We evaluated the accuracy of easily collected parameters obtained before or just after extubation in predicting weaning failure defined as a need for NIV and/or mechanical cough assistance and/or reintubation within 48 h after extubation.

Patients received NIV if they met at least one of the following predefined criteria: respiratory rate > 30 breaths/min; SpO_2_ < 90%; ≥ 20% variation in heart rate or blood pressure; clinical signs of respiratory distress (i.e., cyanosis, sweating, involvement of accessory respiratory muscles, paradoxical abdominal motion, consciousness impairment); PaO_2_ < 60 mm Hg with ≥ 6 L/min O_2_; and hypercapnia with respiratory acidosis (i.e., PaCO_2_ > 45 mm Hg and pH < 7.35). All patients received chest physiotherapy twice daily to promote secretion clearance, with deep inspiration and manual cough assistance. Mechanical cough assistance was used, alone or with NIV, when conventional chest physiotherapy failed to prevent secretion accumulation with severe hypoxemia defined as SaO_2_ < 90% with ≥ 6 L/min O_2_ or FiO_2_ > 50%. Reintubation was considered when there was no improvement within 2 h and was performed according to guidelines [15, 16].

### Statistical analysis

Quantitative variables were described as mean ± SD and qualitative variables as number (%). To compare demographics, clinical data, and LFT results between groups with and without weaning failure as defined above (NIV and/or mechanical cough assistance and/or reintubation, within 48 h after extubation), we used the Chi-square test for categorical variables and the Wilcoxon *t* test for quantitative co-variables. Multivariate logistic regression was performed to identify pre-extubation measurements independently associated with weaning failure. The close correlations among respiratory parameters precluded the use of a single multivariate model. Therefore, we built a separate multivariate logistic regression model to assess the ability of each LFT variable to predict weaning failure. All models were adjusted for MV duration (< 7 vs. ≥ 7 days), MRC scale score (< 48 vs. ≥ 48), and previous chronic respiratory failure. Model discrimination was assessed by the concordance index (c-index) and plotted on a receiver operating characteristic (ROC) curve. For each LFT variable, we identified the cutoff that maximized the Youden index, and we computed the sensitivity and specificity of this cutoff for predicting weaning failure. In addition, correlations between each LFT parameter before and after extubation were assessed by Pearson’s correlation coefficient.

All *P* values were two-tailed with no adjustment for multiple comparisons. *P* values < 0.05 were considered significant. The statistical analyses were performed using SAS statistical software, version 9.4 (SAS Institute Inc., Cary, NC, USA).

## Results

### Study population

**Fig. 1:**
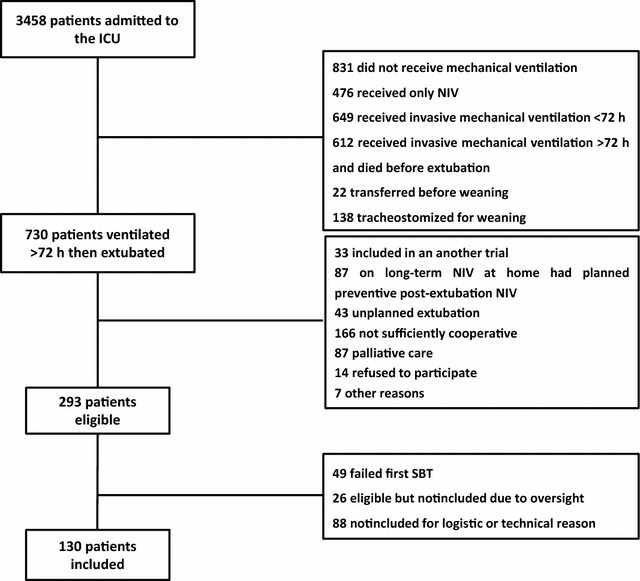
Flowchart of the study

Among 3458 patients admitted to the study ICUs, 730 received MV for more than 72 h and were then extubated; among these, 130 were included in the study (Fig. 1). Table 1 reports their main characteristics at ICU admission. At study inclusion, mean values for ICU stay and MV duration were both 11.0 ± 4.2 days. Five patients were excluded from the analysis because they required immediate reintubation due to either laryngeal edema (*n* = 3) or acute coma (*n* = 2) and consequently could not undergo post-extubation testing.

**Table 1:** Characteristics of the patients at ICU admission

Parameters	Mean ± SD or *n* (%)
Total (*n* = 130)	
Age (years)	59.4 ± 15.6
Male	71 (54.6)
BMI	27.2 ± 6.7
Chronic disease	
Chronic obstructive pulmonary disease	16 (12.3%)
Chronic restrictive pulmonary disease	11 (8.4%)
Chronic heart disease	13 (10%)
SAPS II	45 ± 21
SOFA	7 ± 5
Main reason for ICU admission	
Acute respiratory failure	91 (70)
Heart failure	14 (10.8)
Neurologic failure	9 (6.9)
Septic shock	12 (9.2)
Postoperative	1 (0.8)
Other	3 (2.3)

*BMI* body mass index, *SAPS II* Simplified Acute Physiology Score II [30], *SOFA* Sequential Organ Failure Assessment

After extubation, 36 patients required curative NIV, including 7 who also needed mechanical cough assistance, and 8 required only mechanical cough assistance. Reintubation was performed within 2 days after extubation in 6 patients and on day 6 in 1 patient. All reintubated patients received NIV within 2 days following extubation, and none died in the ICU. Patients who were reintubated were significantly younger and had a lower BMI than those who received only NIV and/or mechanical cough assistance.

### Comparison of lung function parameters before and after extubation

Vital capacity (VC), forced vital capacity (FVC), peak expiratory flow (PEF), and PCEF were significantly higher after than before extubation. Maximal inspiratory pressure (MIP) and maximal expiratory pressure (MEP) were significantly higher before than after extubation (all *P* values < 0.001). As shown in Table 2, the pre-extubation and post-extubation values correlated with each other for all variables (all *P* values < 0.0001); the correlation was strongest for FVC (*R* = 0.89).

**Table 2:** Correlations between physiological parameters before and after extubation

	VC Before extubation	FVC Before extubation	MIP Before extubation	MEP Before extubation	PEF Before extubation	PECF Before extubation
VC After extubation						
*R*	0.61					
*P* value	< *0.0001*					
FVC After extubation						
*R*		0.89				
*P* value		< *0.0001*				
MIP After extubation						
*R*			0.70			
*P* value			< *0.0001*			
MEP After extubation						
*R*				0.66		
*P* value				< *0.0001*		
PEF After extubation						
*R*					0.60	
*P* value					< *0.0001*	
PCEF After extubation						
*R*						0.58
*P* value						< *0.0001*

For each parameter, the table shows the correlation coefficient and *P* value

Italics indicate significant data

*VC* vital capacity, *FVC* forced vital capacity, *MIP* maximal inspiratory pressure, *MEP* maximal expiratory pressure, *PEF* peak expiratory flow, *PCEF* peak cough expiratory flow

### Comparison of patients who did (*n* = 44) and did not (81) require NIV or mechanical cough assistance after extubation

As shown in Table 3, the group that required post-extubation NIV or mechanical cough assistance had a significantly higher proportion of patients with chronic respiratory disease, longer ICU stay and MV durations at study inclusion, and lower MRC scores compared to the other group.

**Table 3:** Univariate analyses

Parameters	No NIV or mechanical cough assistance after extubation (*n* = 81)	NIV or mechanical cough assistance after extubation			*P* value*
	Mean ± SD or *n* (%)	All patients (*n* = 44) Mean ± SD or *n* (%)	Patients who required NIV (*n* = 36) Mean ± SD or *n* (%)	Patients who required Mechanical cough assistance (*n* = 8) Mean ± SD or *n* (%)	
Age, years	58.8 ± 14.8	59.8 ± 16.4	59.6 ± 15.7	60.8 ± 20.3	0.71
SOFA at admission	7.7 ± 5	7.2 ± 4.2	7.5 ± 4.1	5.9 ± 4.8	0.59
Coma Glasgow Scale score	15 ± 0	15 ± 0	15 ± 0	15 ± 0	1.00
Chronic respiratory failure	11 (14%)	14 (32%)	14 (39%)	0	*0.015*
Chronic heart disease	10 (12%)	3 (7%)	3 (8%)	0	0.34
Duration of MV, days	12.7 ± 8.8	17.8 ± 15.6	17.4 ± 14.4	19.8 ± 21.2	*0.02*
Diameter of the endotracheal tube, mm	7.5 ± 0.3	7.4 ± 0.3	7.3 ± 0.3	7.6 ± 0.3	0.17
MRC score	51.1 ± 12	43 ± 15.5	43.2 ± 12.2	42.2 ± 12.2	*0.004*
Tracheal aspiration before extubation (*n*/24 h)	7.8 ± 3	7.7 ± 2.7	7.7 ± 2.5	7.6 ± 3.6	0.89
Respiratory rate (breaths/min)	23.2 ± 11.8	24.5 ± 5.6	24.8 ± 5.9	23.4 ± 4.2	0.50
Borg Scale score (/10)	1.9 ± 2.3	2.1 ± 2.2	2 ± 2	2.3 ± 3.5	0.60
PaCO_2_ before extubation	5.0 ± 0.6	5.6 ± 1	5.8 ± 1	4.9 ± 0.7	*0.00007*
VC (mL) before extubation	1574 ± 498	1281 ± 536	1220 ± 513	1558 ± 586	*0.003*
FVC (mL) before extubation	1571 ± 520	1146 ± 457	1121 ± 464	1257 ± 439	*0.00002*
MIP (cmH_2_O) before extubation	37 ± 15	31 ± 15	32 ± 15	26 ± 12	*0.025*
MEP (cmH_2_O) before extubation	53 ± 28	41 ± 24	44 ± 25	30 ± 16	*0.021*
PEF (L/min) before extubation	80 ± 32	62 ± 30	60 ± 29	71 ± 36	*0.004*
PCEF (L/min) before extubation	97 ± 36	72 ± 33	71 ± 33	75 ± 36	*0.0003*
VC (mL) after extubation	1838 ± 637	1364 ± 499	1343 ± 511	1463 ± 464	*0.00017*
FVC (mL) after extubation	1766 ± 554	1284 ± 433	1284 ± 440	1282 ± 441	*0.00003*
MIP (cmH_2_O) after extubation	28 ± 13	23 ± 11	23 ± 11	22 ± 10	*0.07*
MEP (cmH_2_O) after extubation	43 ± 22	29 ± 17	31 ± 17	21 ± 12	*0.002*
PEF (L/min) after extubation	142 ± 77	107 ± 63	109 ± 66	95 ± 47	*0.02*
PCEF (L/min) after extubation	166 ± 76	107 ± 66	110 ± 72	94 ± 39	*0.0001*

Italics indicate significant data

*SOFA* Sequential Organ Failure Assessment, *MRC* Medical Research Council sum score, *PaO*_*2*_ partial pressure of O_2_ in arterial blood, *PaCO*_*2*_ partial pressure of CO_2_ in arterial blood, *FiO*_*2*_ fraction of inspired O_2_, *VC* vital capacity, *FVC* forced vital capacity, *MIP* maximal inspiratory pressure, *MEP* maximal expiratory pressure, *PEF* peak expiratory flow, *PCEF* peak cough expiratory flow, *NS* nonsignificant

*P values compare patients with and without NIV and/or mechanical cough assistance

By univariate analysis, pre-extubation LFT variables significantly associated with post-extubation NIV and/or mechanical cough assistance were PaCO_2_, VC, FVC, MIP, MEP, PEF, and PCEF (Table 2). Post-extubation LFT variables significantly associated with post-extubation NIV and/or mechanical cough assistance were VC, FVC, MEP, PEF, and PCEF (Table 2).

By multivariate logistic regression adjusted for MV duration, MRC score, and the existence of chronic respiratory failure, variables independently associated with post-extubation NIV and/or mechanical cough assistance were VC, FVC, MIP, MEP, PEF, and PCEF (Table 4).

**Table 4:** Multivariate analysis of extubation predictors

Model	Odds Ratio (IC 95%)	*P* value
Model 1 FVC	0.998 (0.997–0.999)	*0.0005*
Model 2 VC	0.999 (0.998–1.000)	*0.0078*
Model 2 MIP	0.973 (0.947–1.000)	*0.05*
Model 3 MEP	0.983 (0.967–0.999)	*0.043*
Model 4 PEF	0.980 (0.965–0.996)	*0.012*
Model 5 PCEF	0.980 (0.967–0.993)	*0.0022*

One separate model was used for each predictor. All the models were used in multivariable analysis adjusting for the duration of mechanical ventilation (< 7-day vs. 7 days or more), chronic respiratory failure (Yes/No) and MRC (< 48 vs. 48 or more). An odds ratio (OR) > 1 signified an increased probability of necessity of mechanical ventilator assistance

Italics indicate significant data

*VC* vital capacity, *FVC* forced vital capacity, *MIP* maximal inspiratory pressure, *MEP* maximal expiratory pressure, *PEF* peak expiratory flow, *PCEF* peak cough expiratory flow

### ROC curve analysis of performance of the independent predictors

**Fig. 2:**
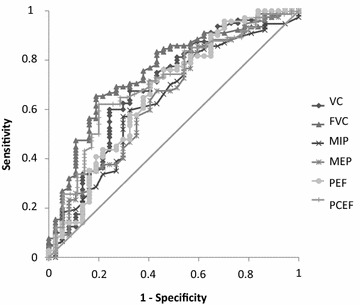
Receiver operating characteristic (ROC) curves for data recorded before extubation: peak cough expiratory flow (PCEF), peak expiratory flow (PEF), forced vital capacity (FVC), slow VC, and maximal inspiratory (MIP) and expiratory (MEP) mouth pressures. AUC, area under the ROC curve

As shown in Fig. 2, the areas under the ROC curves for pre-extubation PCEF, PEF, FVC, MIP, and MEP were 0.71, 0.67, 0.76, 0.61, and 0.69, respectively. The cutoffs that performed best in predicting post-extubation NIV and/or mechanical cough assistance were 85 L/min for PCEF, 62 L/min for PEF, and 1412 mL for FVC. The PCEF cutoff had 74% sensitivity and 62% specificity, the PEF cutoff 51% sensitivity and 76% specificity, and the FVC cutoff 65% sensitivity and 81% specificity.

**Fig. 3:**
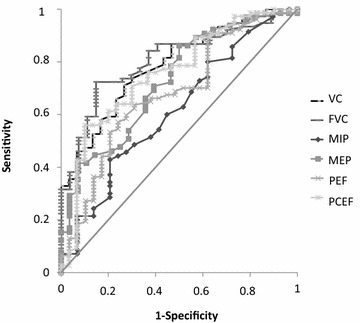
Receiver operating characteristic (ROC) curves for data recorded after extubation: peak cough expiratory flow (PCEF), peak expiratory flow (PEF), forced vital capacity (FVC), slow VC, and maximal inspiratory (MIP) and expiratory (MEP) mouth pressures AUC, area under the ROC curve

As shown in Fig. 3, the areas under the ROC curves for post-extubation PCEF, PEF, FVC, MIP, and MEP were 0.76, 0.68, 0.80, 0.62, and 0.73, respectively. The cutoffs that performed best in predicting post-extubation NIV and/or mechanical cough assistance were 113 L/min for PCEF, 151 L/min for PEF, and 1430 mL for FVC. The PCEF cutoff had 56% sensitivity and 90% specificity, the PEF cutoff 57% sensitivity and 76% specificity, and the FVC cutoff 72% sensitivity and 85% specificity.

## Discussion

The main finding from this study is that the parameter with the closet correlation between pre- and post-extubation values was FVC. FVC may be an objective marker for identifying patients in whom NIV and/or mechanical cough assistance might prevent reintubation. Hypoventilation, cough impairment, and presence of thick and/or excessive mucus can contribute to extubation failure. Most of the previous studies evaluating cough efficiency before extubation focused on PCEF. However, the PCEF cutoffs varied widely [9, 12], perhaps due to differences in study populations and MV durations. Moreover, the diversity of devices used to measure PCEF, presence of a cannula used to bypass the upper airway [19], and differences in the degree of patient coordination and cooperation during measurements may influence the results [12, 20, 21]. In our study, the optimal PCEF cutoff was 85 L/min before extubation and 113 L/min just after extubation. Our pre-extubation PCEF cutoff was higher than in earlier studies. However, our objective was to predict a need for post-extubation NIV and/or mechanical cough assistance, whereas previous studies [12, 20] sought to predict reintubation. Furthermore, the correlation between pre- and post-extubation PCEF values was weak. Several hypotheses can be suggested to explain this finding. The inability of intubated patients to close their glottis limits the pressure generated during coughing and therefore limits the PCEF values compared to those measured without the tube. Also, resistances are higher with than without the endotracheal tube. Finally, in a recent study in tracheostomized patients with neuromuscular disease, PCEF was higher after than before decannulation [13, 22].

Interestingly, Bach and Saporito [7] were the first to use PCEF as a criterion for extubation in patients with neuromuscular disease. However, they measured PCEF immediately after extubation and enhanced performance by combining maximal insufflation with an abdominal thrust timed to glottis opening. The results showed that PCEF > 160 L/min predicted successful extubation. More recently, they challenged their previous PCEF cutoff by demonstrating that professionals who had extensive experience with the noninvasive management of respiratory failure were able to extubate continuously ventilator-dependent patients who had severe cough impairment [8]. Finally, they demonstrated that using noninvasive techniques to improve cough performance and minute ventilation could drastically modify the outcomes of extubated patients, including those dependent on a ventilator [8]. These studies and our data suggest that identifying both the optimal PCEF value and the best PCEF measurement conditions in critically ill patients remains challenging because many factors, including the use of assistive devices, can influence the measurement result.

We tested the usefulness of various LFT parameters for evaluating voluntary cough at the bedside. PCEF and PEF were significantly higher in the successfully extubated group, and low PCEF and PEF values independently predicted post-extubation NIV and/or mechanical cough assistance.

As described previously [23–25], expiratory muscle strength as assessed by the MEP correlated with PCEF. MIP and MEP measurements require a static maneuver with maintenance of a maximal pressure for at least 1.5 s [26]. Nevertheless, contrary to FVC and PCEF, MIP and MEP cannot be measured easily in all mechanically ventilated patients without a specific device.

Our study provides the first evidence that FVC correlates well with PCEF and outperforms PCEF for predicting a need for NIV and/or mechanical cough assistance after extubation. In addition, FVC was the parameter least affected by the presence of a tracheal tube, so that pre-extubation FVC < 1420 mL was 64% sensitive and 81% specific, with improvements to 72 and 85%, respectively, when FVC remained < 1420 mL after extubation. This is not surprising given that FVC diminishes only in the event of air trapping, which is generally due to peripheral airway obstruction and not to increased central airway resistance due, for instance, to a tracheal tube.

Several limitations of our study should be addressed. First, we included only those patients who were sufficiently cooperative and were extubated at a time when the technician was available for pre-extubation LFT. This requirement decreased the number of included patients but allowed the physicians to remain blinded to LFT findings, thereby minimizing bias. Thus, of the 730 patients extubated during the study period, 130 (18%) were included. Second, we did not assess involuntary cough. However, recent work indicates that, in cooperative patients, voluntary PCEF is far more accurate than involuntary PCEF in predicting reintubation, due to underestimation of cough strength by involuntary PCEF in patients with high voluntary PCEF [21]. We deliberately confined our study to cooperative patients, since we used noninvasive but volitional measurement techniques. Third, we excluded patients with MV for less than 72 h, since extubation failure is rare in this situation. Fourth, we did not measure the rapid shallow breathing index or fluid balance, two variables significantly associated with extubation failure in a previous study [27]. However, all study patients passed an SBT. Surprisingly, maximal pressures decreased after extubation, whereas the other parameters increased. This finding may be ascribable to the difference in patient-measurement device interface between pre- and post-extubation [28, 29]. In addition, upper-airway muscle activation and coordination are usually required when using a flanged mouthpiece but are not required when a tracheal tube bypasses the upper airway, which allows the patient to concentrate the effort on the inspiratory or expiratory muscles. Finally, a tracheal tube may diminish airway compliance and, therefore, the volume change during breathing, resulting in higher pressures for the same effort. Fifth, as this study used a prospective observational design, we did not change the practices in each center regarding the use of preventive NIV. The percentage of reintubated patients was surprisingly small in our study, i.e., 3 times lower than in the study by Esteban et al. among patients receiving NIV (48 vs. 16%). This difference may be ascribable to the high prevalence in our study of patients with COPD or restrictive pulmonary disease (20.7%), who may derive particularly large benefits from NIV [30]. Although ERS/ATS guidelines do not recommend using NIV to avoid reintubation in patients with overt respiratory distress and/or respiratory failure after planned extubation, this recommendation is not considered definitive and may not apply to patients with COPD [31]. Furthermore, reported benefits of curative NIV include improved oxygenation and alveolar ventilation, better alveolar recruitment in patients with atelectasis, improved left ventricular function in patients with heart failure, and decreases in intrinsic PEEP and work of breathing [32].

A legitimate issue is whether postponing extubation might have decreased the reintubation rate in our patients, who had longer MV durations before extubation compared to those in recent studies [5, 33, 34]. This difference is due to the inclusion in our study of only those patients already on MV for 72 h. However, our patients were extubated as soon as the daily conventional SBT was successful, in keeping with recent guidelines about the optimal assessment of weaning readiness [35].

Another factor that may have contributed to the low reintubation rate in our population is the considerable experience of our staff in the noninvasive treatment of patients with chronic and complete ventilator dependency [36–38]. We share this high level of experience with teams specialized in neuromuscular diseases [39]. Moreover, the addition to NIV of mechanical insufflation-exsufflation when appropriate may have further decreased the reintubation needs, as shown in a recent randomized trial [40]. Given the persistent challenges in identifying patients at high risk of post-extubation respiratory failure requiring, at best, NIV or mechanical cough assistance and, at worst, reintubation, we chose weaning failure defined as the use of NIV, cough assistance, and/or reintubation as the study endpoint.

Finally, as demonstrated by Thille et al. [41] the ability of healthcare staff to predict extubation failure is poor. The results reported here should help to identify patients likely to benefit from preventive NIV or cough assistance, using simple physiological parameters. These results need to be confirmed in a large epidemiological study including clinical and physiological variables [33].

## Conclusion

In conclusion, our main finding is that FVC measurements before and after extubation are well correlated. FVC may serve as an objective predictor of post-extubation respiratory failure requiring NIV and/or mechanical cough assistance and/or reintubation in heterogeneous populations of medical ICU patients. FVC measurement may deserve consideration as an inexpensive tool to be used in combination with easily identified risk factors for assessing patients after a successful SBT, with the goal of identifying those likely to require prophylactic post-extubation NIV and/or mechanical cough assistance. However, further studies are necessary to confirm our results in different conditions and populations.

## Additional file

**Additional file 1:** Details on methods.

## Abbreviations

FVC: Forced vital capacity
ICU: Intensive care unit
LFT: Lung function testing
MEP: Maximal expiratory pressure
MIP: Maximal inspiratory pressure
MRC sum score: Medical Research Council sum score
MV: Endotracheal mechanical ventilation
NIV: Noninvasive ventilation
PaO_2_: Partial pressure of O_2_ in arterial blood
PaCO_2_: Partial pressure of CO_2_ in arterial blood
PCEF: Peak cough expiratory flow
PEF: Peak expiratory flow
ROC curve: Receiver operating characteristic curve
SBT: Spontaneous breathing trial
SOFA: Sequential Organ Failure Assessment
VC: Vital capacity

## References

[1] Epstein SK (2002). Decision to extubate. Intensive Care Med.

[2] Esteban A, Anzueto A, Frutos F, Alia I, Brochard L, Stewart TE, Benito S, Epstein SK, Apezteguia C, Nightingale P, Arroliga AC, Tobin MJ (2002). Characteristics and outcomes in adult patients receiving mechanical ventilation: a 28-day international study. JAMA.

[3] Esteban A, Frutos F, Tobin MJ, Alia I, Solsona JF, Valverdu I, Fernandez R, de la Cal MA, Benito S, Tomas R, Carriedo D, Macias S, Blanco J (1995). A comparison of four methods of weaning patients from mechanical ventilation. Spanish Lung Failure Collaborative Group. N Engl J Med.

[4] Thille AW, Cortes-Puch I, Esteban A (2013). Weaning from the ventilator and extubation in ICU. Curr Opin Crit Care.

[5] Thille AW, Harrois A, Schortgen F, Brun-Buisson C, Brochard L (2011). Outcomes of extubation failure in medical intensive care unit patients. Crit Care Med.

[6] Vallverdu I, Calaf N, Subirana M, Net A, Benito S, Mancebo J (1998). Clinical characteristics, respiratory functional parameters, and outcome of a two-hour T-piece trial in patients weaning from mechanical ventilation. Am J Respir Crit Care Med.

[7] Bach JR, Saporito LR (1996). Criteria for extubation and tracheostomy tube removal for patients with ventilatory failure. A different approach to weaning. Chest.

[8] Bach JR, Goncalves MR, Hamdani I, Winck JC (2010). Extubation of patients with neuromuscular weakness: a new management paradigm. Chest.

[9] Beuret P, Roux C, Auclair A, Nourdine K, Kaaki M, Carton MJ (2009). Interest of an objective evaluation of cough during weaning from mechanical ventilation. Intensive Care Med.

[10] Khamiees M, Raju P, DeGirolamo A, Amoateng-Adjepong Y, Manthous CA (2001). Predictors of extubation outcome in patients who have successfully completed a spontaneous breathing trial. Chest.

[11] Su WL, Chen YH, Chen CW, Yang SH, Su CL, Perng WC, Wu CP, Chen JH (2010). Involuntary cough strength and extubation outcomes for patients in an ICU. Chest.

[12] Smina M, Salam A, Khamiees M, Gada P, Amoateng-Adjepong Y, Manthous CA (2003). Cough peak flows and extubation outcomes. Chest.

[13] McKim DA, Hendin A, LeBlanc C, King J, Brown CR, Woolnough A (2012). Tracheostomy decannulation and cough peak flows in patients with neuromuscular weakness. Am J Phys Med Rehabil.

[14] McCool FD (2006). Global physiology and pathophysiology of cough: ACCP evidence-based clinical practice guidelines. Chest.

[15] Perren A, Brochard L (2013). Managing the apparent and hidden difficulties of weaning from mechanical ventilation. Intensive Care Med.

[16] Boles JM, Bion J, Connors A, Herridge M, Marsh B, Melot C, Pearl R, Silverman H, Stanchina M, Vieillard-Baron A, Welte T (2007). Weaning from mechanical ventilation. Eur Respir J.

[17] Kress JP, Hall JB (2014). ICU-acquired weakness and recovery from critical illness. N Engl J Med.

[18] Borg GA (1982). Psychophysical bases of perceived exertion. Med Sci Sports Exerc.

[19] Lofaso F, Louis B, Brochard L, Harf A, Isabey D (1992). Use of the Blasius resistance formula to estimate the effective diameter of endotracheal tubes. Am Rev Respir Dis.

[20] Salam A, Tilluckdharry L, Amoateng-Adjepong Y, Manthous CA (2004). Neurologic status, cough, secretions and extubation outcomes. Intensive Care Med.

[21] Duan J, Liu J, Xiao M, Yang X, Wu J, Zhou L (2014). Voluntary is better than involuntary cough peak flow for predicting re-intubation after scheduled extubation in cooperative subjects. Respir Care.

[22] Kang SW, Choi WA, Won YH, Lee JW, Lee HY, Kim DJ (2016). Clinical Implications of Assisted Peak Cough Flow Measured with an External Glottic Control Device for Tracheostomy Decannulation in Patients with Neuromuscular Diseases and Cervical Spinal Cord Injuries: A Pilot Study. Arch Phys Med Rehabil.

[23] Mahajan RP, Singh P, Murty GE, Aitkenhead AR (1994). Relationship between expired lung volume, peak flow rate and peak velocity time during a voluntary cough manoeuvre. Br J Anaesth.

[24] Suleman M, Abaza KT, Gornall C, Kinnear WJ, Wills JS, Mahajan RP (2004). The effect of a mechanical glottis on peak expiratory flow rate and time to peak flow during a peak expiratory flow manoeuvre: a study in normal subjects and patients with motor neurone disease. Anaesthesia.

[25] Park JH, Kang SW, Lee SC, Choi WA, Kim DH (2010). How respiratory muscle strength correlates with cough capacity in patients with respiratory muscle weakness. Yonsei Med J.

[26] American Thoracic Society/European Respiratory Society (2002). ATS/ERS statement on respiratory muscle testing. Am J Respir Crit Care.

[27] Frutos-Vivar F, Ferguson ND, Esteban A, Epstein SK, Arabi Y, Apezteguia C, Gonzalez M, Hill NS, Nava S, D’Empaire G, Anzueto A (2006). Risk factors for extubation failure in patients following a successful spontaneous breathing trial. Chest.

[28] Montemezzo D, Vieira DS, Tierra-Criollo CJ, Britto RR, Velloso M, Parreira VF (2012). Influence of 4 interfaces in the assessment of maximal respiratory pressures. Respir Care.

[29] Koulouris N, Mulvey DA, Laroche CM, Green M, Moxham J (1988). Comparison of two different mouthpieces for the measurement of Pimax and Pemax in normal and weak subjects. Eur Respir J.

[30] Peter JV, Moran JL, Phillips-Hughes J, Warn D (2002). Noninvasive ventilation in acute respiratory failure—a meta-analysis update. Crit Care Med.

[31] Rochwerg B, Brochard L, Elliott MW, Hess D, Hill NS, Nava S, Navalesi PMOTSC, Antonelli M, Brozek J, Conti G, Ferrer M, Guntupalli K, Jaber S, Keenan S, Mancebo J, Mehta S, Raoof SMOTTF (2017). Official ERS/ATS clinical practice guidelines: noninvasive ventilation for acute respiratory failure. Eur Respir J.

[32] Vitacca M, Ambrosino N, Clini E, Porta R, Rampulla C, Lanini B, Nava S (2001). Physiological response to pressure support ventilation delivered before and after extubation in patients not capable of totally spontaneous autonomous breathing. Am J Respir Crit Care Med.

[33] Thille AW, Boissier F, Ben-Ghezala H, Razazi K, Mekontso-Dessap A, Brun-Buisson C, Brochard L (2016). Easily identified at-risk patients for extubation failure may benefit from noninvasive ventilation: a prospective before-after study. Crit Care (London, England).

[34] Beduneau G, Pham T, Schortgen F, Piquilloud L, Zogheib E, Jonas M, Grelon F, Runge I, Nicolas T, Grange S, Barberet G, Guitard PG, Frat JP, Constan A, Chretien JM, Mancebo J, Mercat A, Richard JM, Brochard L, Group WS, The RNdd (2017). Epidemiology of weaning outcome according to a new definition. The WIND Study. Am J Respir Crit Care Med.

[35] Quintard H, l’Her E, Pottecher J, Adnet F, Constantin JM, De Jong A, Diemunsch P, Fesseau R, Freynet A, Girault C, Guitton C, Hamonic Y, Maury E, Mekontso-Dessap A, Michel F, Nolent P, Perbet S, Prat G, Roquilly A, Tazarourte K, Terzi N, Thille AW, Alves M, Gayat E, Donetti L (2017). Intubation and extubation of the ICU patient. Anaesth Crit Care Pain Med.

[36] Nardi J, Leroux K, Orlikowski D, Prigent H, Lofaso F (2016). Home monitoring of daytime mouthpiece ventilation effectiveness in patients with neuromuscular disease. Chronic Respir Dis.

[37] Lacombe M, Del Amo Castrillo L, Bore A, Chapeau D, Horvat E, Vaugier I, Lejaille M, Orlikowski D, Prigent H, Lofaso F (2014). Comparison of three cough-augmentation techniques in neuromuscular patients: mechanical insufflation combined with manually assisted cough, insufflation-exsufflation alone and insufflation-exsufflation combined with manually assisted cough. Respir Int Rev Thorac Dis.

[38] Lofaso F, Prigent H, Tiffreau V, Menoury N, Toussaint M, Monnier AF, Stremler N, Devaux C, Leroux K, Orlikowski D, Mauri C, Pin I, Sacconi S, Pereira C, Pepin JL, Fauroux B, Association Francaise Contre les Myopathies research g (2014). Long-term mechanical ventilation equipment for neuromuscular patients: meeting the expectations of patients and prescribers. Respir Care.

[39] Bach JR (2017). Noninvasive respiratory management of patients with neuromuscular disease. Ann Rehabil Med.

[40] Goncalves MR, Honrado T, Winck JC, Paiva JA (2012). Effects of mechanical insufflation-exsufflation in preventing respiratory failure after extubation: a randomized controlled trial. Crit Care (London, England).

[41] Thille AW, Boissier F, Ben Ghezala H, Razazi K, Mekontso-Dessap A, Brun-Buisson C (2015). Risk factors for and prediction by caregivers of extubation failure in ICU patients: a prospective study. Crit Care Med.

