# Supplementary material for: Physiological predictors of respiratory and cough assistance needs after extubation
Source: Ann Intensive Care. 2018 Feb 5;8:18. doi: 10.1186/s13613-018-0360-3 (PMC5799095; doi:10.1186/s13613-018-0360-3)
Supplement: Supplementary file 1 — Additional file 1: Details on methods. [file 13613_2018_360_MOESM1_ESM.docx]

**Physiological Predictors of Respiratory and Cough Assistance Needs after Extubation**

Nicolas TERZI, MD, PhD; Frédéric LOFASO, MD, PhD; Romain MASSON, MD; Pascal BEURET, MD; Hervé NORMAND, MD, PhD; Edith DUMANOWSKI; Line FALAIZE; Bertrand SAUNEUF, MD; Cédric DAUBIN, MD; Jennifer BRUNET, MD; Djillali ANNANE, MD, PhD;Jean-Jacques PARIENTI, MD, PhD and David ORLIKOWSKI, MD, PhD

Online data supplement

**METHODS**

## Lung function testing (LFT)

Spirometry and respiratory muscle strength testing were performed by an experienced technician who was not otherwise involved in the study. During testing, patients were seated in bed. Patients were allowed time to recover fully between tests. Spirometry was performed according to standard guidelines [[1](#_ENREF_1)]. The spirometer (Spirodoc^®^, Medical International Research, Rome, Italy) and respiratory pressure meter (MicroRPM^®^, CareFusion, San Diego, CA, USA) were connected to the proximal end of the endotracheal tube. Maximal inspiratory pressure (MIP) was measured from functional residual capacity and maximal expiratory pressure (MEP) from total lung capacity. Slow vital capacity (VC) and forced vital capacity (FVC) were recorded, as well as peak expiratory flow (PEF) during the FVC maneuver and peak cough expiratory flow (PCF) during a cough maneuver. Pressure measurements were repeated at least three times or until two identical readings were obtained [[2](#_ENREF_2)]; the best value of three maneuvers that varied by less than 20% was recorded [[2](#_ENREF_2)] and expressed as the absolute value in cmH_2_O. Arterial blood gas values were measured (Radiometer ABL 330, Tacussel, Copenhagen, Denmark).

**REFERENCES**

1. Quanjer PH, Tammeling GJ, Cotes JE, Pedersen OF, Peslin R, Yernault JC (1993) Lung volumes and forced ventilatory flows. Report Working Party Standardization of Lung Function Tests, European Community for Steel and Coal. Official Statement of the European Respiratory Society. The European respiratory journal Supplement 16:5-40.

2. Wilson SH, Cooke NT, Edwards RHT, Spiro SG (1984) Predicted normal values for maximal respiratory pressures in caucasian adults and children. Thorax 39:535-8.
